# The role of self-efficacy and assertiveness in aggression among high-school students in Isfahan

**Published:** 2015

**Authors:** SH Khademi Mofrad, T Mehrabi

**Affiliations:** * Student Research Center, School of Nursing and Midwifery, Isfahan University of Medical Science; ** Nursing and Midwifery Faculty, Isfahan University of Medical Science

**Keywords:** aggression, assertiveness, self-efficacy, high school students, Iran

## Abstract

**Background.** Nowadays, one sixth of the world’s population is represented by adolescents, nearly 1.2 billion people being of age 10-19. According to the 2011 census in Iran, the estimation of adolescent population was 12 million, which represents 16% of the Iran population. Undoubtedly, adolescence is the most dominant stage of life. During this period, adolescents face biological, cognitive, and emotional changes that may be accompanied by inappropriate behavioral responses such as aggression. Considering pressures of peer groups during adolescence, assertiveness has an important role as a social skill. It seems that the success of adolescents in dealing with these problems depends on their self-efficacy. This study was designed to explore the role of self-efficacy and assertiveness in aggression among high-school students.

**Material and methods.** This cross-sectional and correlational study was conducted among 321 first grade high-school students during 2014 and 2015. Samples were extracted from six education and training regions by a multi-stage random sampling. In this study, the questionnaire included demographic, Rathus Assertiveness, self-efficacy for children and aggression data.

**Results. ** The results showed that there was a notable negative association between aggression and assertiveness (p < 0.003) and also between assault and self-efficacy (p < 0.001).

**Conclusions.** An increase in assertiveness and self-efficacy resulted in a decrease of aggression. So, training was recommended to reinforce self-efficacy beliefs and assertiveness behaviors for mental health promotion.

## Background

Today, one sixth of the world’s population is represented by adolescents. In other words, at least 1.2 billion people have ages between 10 and 19 [**1**]. The Iran 2011 National Population and Housing Census estimated that there are twelve million adolescents, that is, up to 16% of the population [**2**]. Half of all the adulthood mental disorders start at the age of 14, most of them remaining unknown, no treatment being administered [**1**]. This period is undoubtedly the most significant stage of the human life. Adolescence could be called a crisis period because of the maturity problems and the complexity of life [**3**]. In other words, adolescence may be considered the transitional stage from childhood to adulthood. In addition to dealing with biological, cognitive, emotional, and moral changes, the adolescent has to live up to the expectations of parents, school and peers [**4**] and coping with these forms of problems could be stressful. Another critical stage is the attraction between teenagers (same & hetero-sex). At this point, the adolescent may enter the environment in which there is less personal support and supervision [**5**]. According to many experts, during this period, adolescents experience nearly as many needs and conflicts that are often associated with undesirable behavioral responses. Aggression is one of these answers [**6**]. Human aggression is any action directly intended to harm one person by another [**7**-**9**]. Nowadays, two critical theories about aggression are accepted in the domain of social behavior. In Freud’s psychoanalytic theory, the attack is considered one of the drives, while in social learning theory, aggression is a learned response [**10**]. Aggression can take a variety of forms that may be physically expressed or communicated verbally or non-verbally: the verbal and physical attacks include behavioral aspects, anger representing an emotional or affective component, hostility being the cognitive dimension of aggression [**11**]. The more obvious and problematic face of aggression can be seen in adolescents due to environmental and biochemical changes occurring in the body [**12**]. Farrell et al. believe that the school, peers, and parents play an important role in the violence growth in the time of transition from childhood to early adolescence. Peers attitudes are imperative, especially in the transition from the last stage of primary school to the first stage of high school. Also on this stage, the school circumstances may intensify violence. In this period, teenagers may have discussions, debates, and sometimes, inappropriate behaviors to demonstrate maturity as well as to improve their social status in the peers network [**13**]. Researchers focus on studying the aggressive behaviors during this period, because of the outcomes such as the negative image among peers and teachers, bad relationships with the family, school failure, rejection by peers and delinquency [**14**].

Of course, the root of tensions in schools can be found in the interpersonal communication. Students are usually from different cultural and social backgrounds and lack education [**15**]. One of the social skills that plays an important role in the interpersonal communication is assertiveness. Assertiveness is a type of relationship that helps people protect their rights without underestimating and attacking the rights of others as well as express thoughts, feelings, and opinions in a clear, honest and appropriate way [**16**]. It seems that this skill is important during this period (adolescence) due to the pressure from peers and also their age circumstance [**3**].

During this time, adolescents are faced with new challenges and are exposed to many social traumas. Studies showed that teenage success in dealing with problems of this area is highly associated with their self-efficacy [**17**]. According to Bandura’s theory, self-efficacy refers to the beliefs regarding one’s capabilities of successfully completing tasks [**18**]. The theory points out that students form their self-efficacy beliefs by interpreting data from four references. The most prominent reference is the interpreted result of one’s previous performance or mastery experience that enables students a successful completion of tasks. In addition to interpreting the outcomes of their actions, people create their self-efficacy beliefs through the vicarious experience of observing the others. Individuals then also promote self-efficacy beliefs as a result of social persuasions they receive from others as well as physical and emotional states such as anxiety, stress, arousal, and mood states [**19**].

Social, educational, emotional, and physical scopes are amongst the most important elements of the adolescent’s self-efficacy [**20**].

Previous studies lacked a description of aggression in adolescents, resulting in the formation of unhealthy relationships in school as well as causing consequences such as truancy, substance abuse, and academic failure, which is an obstacle in the prosperity of their empowerment and socialization [**21**]. The World Health Organization has revealed that nearly 180 teens die due to interpersonal violence every day [**1**]. Violence sharply increases the cost of health, welfare, and judicial services in the field of crime and, overall, causes the weakness of the structure of society [**22**]. Given the prevalence of 30 to 50 percent of aggression cases in Iranian adolescents [**23**] and its costs and consequences, it is important to identify some of the factors associated with it.

A review of studies showed that there is a considerable amount of literature on the psychological constructs such as assertiveness, self-efficacy, and aggression, but there have been a few controlled studies, which determined the role of assertiveness and self-efficacy and its dimensions on the students’ attack. This study aimed to determine the effect of these variables, which have a significant role in any adolescent’s mental health. 

## Materials and Methods

The present cross-sectional study was a descriptive–analytic survey designed to explore the role of self-efficacy and assertiveness in aggression among 321 high-school students (boys and girls) in Isfahan during the academic year 2014-2015.

The criteria of adolescent inclusion were the following: having no mental disorders, lacking the use of psychedelic drugs, no smoking, and no separation of their parents, as well as having parents alive and living with them. The respondents were selected by multistage random sampling. From the selected high schools, a class was randomly selected until the total number of students rose to 341 and then, taking into account the entrance criteria, it was reduced to 321. 

In this study, the demographic information questionnaire and the assertiveness, self-efficacy, and aggression questionnaire were used. The demographic data questionnaire included demographic variables such as age, gender, GPA for years ago, the number of siblings, parents’ education, and parents’ occupation. The Rathus Assertiveness Schedule contained 30 questions. The questionnaire scores fluctuated between +90 (very high assertiveness) to -90 (lack of assertiveness). In his study conducted in 1998, Bahraini found a correlation (r = 0.78), for the Rathus Assertiveness Schedule [**24**]. In the Schedule, students with a total score of less than +10 meant they were shy and nonassertive, and scores above +10 meant they were assertive [**25**]. Aggression was measured by using the 30 questions aggressive Questionnaire (AGQ). 14 items measured the anger, eight questions invasion, and eight questions malice. The scores more than the average of 42.5 in this test presented a more competitive rate. In 1996, Najarian calculated the validity of the Aggression Questionnaire at 0.85 [**26**].

Each student’s score for self-efficacy was obtained by the Self-Efficacy Questionnaire for Children (SEQ-C). The Self-Efficacy Questionnaire for Children (SEQ-C; Muris, 2001) assessed the general self-efficacy across three domains: social, academic, and emotional situations. The SEQ-C is a 23 items self-report measure with eight objects for social, eight items for academic and seven items for the emotional domain. Each item is rated on a five-point Likert scale with one being “not at all” and five being “very well”. 

The questionnaire’s reliability was shown to be good and its internal consistency was calculated at 0.80. The reliability of the SEQ-C was reported to be 0.78, 0.87, and 0.80 for the social, academic, and emotional subscales, respectively [**20**,**27**].

The data were analyzed by using Pearson correlation, Spearman, independent t-test, variance analysis, and regression in IBM SPSS Statistics version 20 software at a significance level of 0.05.

## Results 

In this study, the average age of the students was 15.39 ± 0.49: 148 girls (46.1%) and 173 boys (53.9%). The GPA was 17.54 ± 2.03. While reviewing the parent’s occupation element, it was shown that most of the fathers were self-employed (34.3%), whereas the majority of the mothers were households (89.4%). Also, the results showed that most of the fathers had guidance school grade (23.9%) and most of the mothers had high school diplomas (26.2%).

According to the study, the mean score of aggression was 41.54 ± 13.26, assertiveness 4.05 ± 18.75 and self-efficacy 79.38 ± 11.37 (**Table 1**). The attack score of 56.6% of the students was lower than the cut-off point and 43.4% higher than the cut-off point of aggressiveness. Also, 48.3% of the students had scores above-average regarding self-efficacy and in terms of assertiveness 66.5% of them were shy and nonassertive, as well as 33.5% of them were assertive.

**Table 1:** Conventional and standard deviation (SD) of aggression, assertiveness, self-efficacy, and their subscales

variables	Mean	SD
Aggression	41.54	13.26
Anger	23.49	6.21
Invasion	7.95	4.59
Malice	10.48	5.02
Assertiveness	4.05	18.75
Self-Efficacy	79.38	11.37
Social	27.81	4.78
Educational	29.67	4.98
Emotional	21.78	5.21

p = 0.003, r = -0.17) and the self-efficacy and aggression scores (p < 0.001, r = -0.349). In other words, the more self-efficacy and assertiveness the students have, the less aggressive they are. Proceeds of multiple regression examinations with adjustment for gender, parents education, and GPA showed that assertiveness in reverse (B = -0.085, p = 0.039) as well as self-efficacy in reverse (B = -0.355, p < 0.001) are significant predictor variables for the aggression. It is worth noting that in predicting the aggressiveness score, self-efficacy was a more powerful predictor than the assertiveness (**Table 2**,**3**).

**Table 2:** Result of multiple regression of ASQ and individual variables on aggression R2 = 0.086, F = 5.537, p-value < 0.001

variables	B	SE	Beta	p-value
Sex	-1.932	1.513	-0.073	0.203
Mother’s education	-1.235	0.795	-0.123	0.122
Father’s education	0.479	0.848	0.046	0.572
Average	-1.288	0.417	-0.195	0.002
ASQ	-0.085	0.041	-0.119	0.039

**Table 3:** Result of multiple regression of SEQ and individual variables on aggression R2 = 0.155, F=10.99, p-value < 0.001

variables	B	SE	Beta	p-value
Sex	-0.569	1.474	-0.021	0.7
Mother’s education	-1.027	0.756	-0.102	0.175
Father’s education	0.248	0.809	0.024	0.76
Average	-0.829	0.411	-0.125	0.045
SEQ	-0.355	0.067	-0.304	<0.001

Also, the results showed that there was an inverse relationship between the score of aggression with self-efficacy dimensions including academic (p < 0.001, r = - 0/ 315), emotional (p < 0.001, r = -0.304) and social (p < 0.006, r = - 0.154) domains. In other words, students who had a higher aggressiveness were lower in social, academic, and emotional self-efficacy. In multiple regression analysis, when the impacts of social, academic and emotional self-efficacy on aggression were simultaneously reviewed, academic (B = -0.717, p < 0.001) and psychological dimensions (B = -0.559, p < 0.001) were significant but the social aspect (B = 0.09, p = 0.581) was not significant (**Table 4**).

**Table 4:** Regression analysis between self-efficacy subscales and aggression R2 = 0.151, F = 18.323, p-value < 0.001

variables	B	SE	Beta	p-value
Social	0.09	0.163	0.033	0.581
Educational	-0.717	0.159	-0.27	< 0.001
Emotional	-0.559	0.144	-0.221	< 0.001

In the relationship between GPA and the total scores for aggression, self-efficacy and assertiveness, the results showed that there was an inverse relation between GPA and the overall scores for aggression (p < 0.001, r = - 0.235). Whereas, there was a direct relationship between GPA and the total score for self-efficacy (p < 0.001, r = - 0.269) as well as between GPA and the assertiveness score (p < 0.001, r = - 0.188).

There was an inverse relation between the parents’ education and the aggression rating, (p < 0.05) but there was a significant direct correlation between the parents’ education and the self-efficacy score and also the assertiveness score (p < 0.05). The mothers’ education had a stronger relationship with these variables (**Table 5**).

**Table 5:** Pearson Correlation (r) between the parents’ education and aggression, self-efficacy, assertiveness

Variable	Father’s education		Mother’s education	
	r	p-value	r	p-value
Aggression	-0.15	0.008	-0.194	0.001
Self-efficacy	0.203	<0.001	0.222	<0.001
Assertiveness	0.222	<0.001	0.244	<0.001

Also, the independent t-test showed that the self-efficacy scores were higher in boys than in girls (p <0.05). However, there was no meaningful difference between the boys and girls scores for the assertiveness (p > 0.05) and aggression (p > 0.05) (**Table 6**).

**Table 6:** Mean values of aggression, self-efficacy, and assertiveness among girls and boys

variables	Girls		Boys		Independent t-test	
	Mean	SD	Mean	SD	Mean	SD
Aggression	42.30	13.20	40.9	13.3	0.99	0.321
Self-efficacy	77.90	12.40	80.6	10.3	2.00	0.040
Assertiveness	2.90	19.94	5.03	17.67	-1.00	0.318

ANOVA demonstrated that there was a meaningful relationship between the father’s occupation and aggression (p < 0.05) and the assertiveness scores (p < 0.05) of students. However, there was not a significant association between the father’s profession and the student self-efficacy (p > 0.05). Further studies showed that there was a significant relationship between the father’s occupation and the academic area (p < 0.05).

Also, the ANOVA test showed that there was not a significant association between the mother’s occupation and aggression (p > 0.05) and the self-efficacy scores for students (p > 0.05), but a significant association between the mother’s occupation and the assertiveness of students (p < 0.05) was observed. 

## Discussion 

The results showed that with increasing assertiveness and self-efficacy, aggression would be reduced. Researchers believe that certain people act fair and in a reasoning manner and are committed to mutual respect in social communication, also having appropriate skills in resolving the conflict. Thus, outcomes in aggressive behavior are seen less [**27**]. These findings were consistent with the results of other studies, including Khazaee and colleagues, Khalatbary and colleagues and Ashuri and colleagues [**27**-**29**].

In this study, students who enjoyed a higher self-efficacy had lower aggression. Tahmasian and Ansari suggested that people who obtain a high self-efficacy, have an accurate knowledge of their abilities, enjoy a real social connection, and are capable of controlling and managing their emotions, in addition, they show a less aggressive behavior [**30**]. On the other hand, individuals with low self-efficacy may perceive the events to be more complicated than the fact that leads to increased stress and anxiety [**31**].

The results showed that students who had a high academic, emotional, and social self-efficacy, had less aggressive behaviors too. It is necessary to consider that aggressive people have no management on how to express their emotions [**27**], while self-efficacy leads to an ability to manage emotions [**32**].

The results of this study were consistent with Sayapoor and colleagues’ study, in which they found that students with higher scores on aggression had a less academic self-efficacy [**1**].

In this study, students who had a higher GPA were less aggressive. Other researchers showed a significant direct relation between aggression and learning difficulties so that one-quarter of children with Bradylexia and one-third of children with a reading disability were aggressive [**15**]. Furthermore, the study results were consistent with those of Shojaee and colleagues who aimed to determine the predictors of aggression in adolescents in the city of Qom [**33**].

Another finding of this study showed that there was a direct relationship between the GPA and the total score of self-efficacy. The result was in line with the results of Askari and colleagues. The results showed that there was a significant relationship between self-efficacy and academic performance [**34**]. Bandura believes that learners with a high self-efficacy show a better performance in educational activities in the face to challenges, than the people who doubt their capabilities [**35**].

Results showed that there was a direct relationship between assertiveness scores and GPA. The study was consistent with the results of Mehrabizadeh and colleagues who showed that training assertiveness led to the reducing of social anxiety as well as to the increase of social skills and academic performance of students in the experimental group [**36**]. Also, the higher education grades parents had, the more assertive scores students acquired.

Results showed that there is a stronger relationship between the variables mentioned above and the mothers’ education than the father’s education. Various theories suggested that the security of the mother-child relationship could cause intimate relationships, self-esteem, and mental evolution on the long term [**37**]. Also, studies have shown that parents with higher education levels, higher social and economic statuses, are appropriate models for their children and generate a more positive verbal relationship with the kids that leads to increased self-efficacy in children. Such parents also benefit from more communication and use better-coping strategies to control emotions that are a cause of the inverse relationship between education and the implications of aggression [**17**]. Moreover, the present study was in line with Mohebi and colleagues’ research, which showed that parents with higher education have more firm adolescents [**3**].

The results of the average scores for aggression, self-efficacy, and assertiveness of girls and boys showed that the total scores of self-efficacy in boys were higher than in girls. This study was consistent with the Mirghafurv and colleagues’ study. This study suggested that a possible reason for the higher rate of self-efficacy in boys, is related to culture and social norms, resulting in playing more prominent personal and social roles for boys than girls and thus more confidence and higher self-efficacy in males was registered [**38**]. In this study, male and female students were not significantly different regarding aggression and assertiveness. The research results were in line with the results of Golchin [**39**], Shafiee and Safari Nia [**40**]. Golchin admitted that more restrictions for girls compared to boys probably leads to their increased aggression [**39**].

The results showed that there was a significant relationship between the father’s occupation and the student’s aggression/ assertiveness scores. The aggression of the students whose fathers were clerks is lower than of the others, but their assertiveness score is higher [**26**]. Perhaps it can be concluded from this study that the parents who are employees have a greater ability to meet social, economic, and academic needs of their children in the proper manner. As a result, they have trained children with less aggression and higher assertiveness. It is necessary to consider that there was no significant relationship between the mother’s occupation and the students’ aggression. Given that 89.4 percent of mothers were homemakers, this figure influenced the results.

Also, the results showed that there was no significant relationship between the self-efficacy score and the parent’s occupation. In a study, with results inconsistent with the present study, Mirghafurv and colleagues concluded that the father’s occupation was a variable that could work as a predictor of self-efficacy in adolescents. In their study, adolescents whose fathers were unemployed a lower self-efficacy score than teenagers whose parents were self-employed [**38**]. The differences may be explained in the level of education, self-efficacy scale and the inclusion criteria of the study. 

Therefore, the researchers decided to examine the relationship between parental occupations with the self-efficacy. The results showed a significant correlation only between the father’s occupation, and the academic domain of self-efficacy. In their study, Dehghani and Hosseinchari revealed that families pay less attention to the social and emotional dimensions of their children self-efficacy than to the academic one. In other words, transferring positive self-concept beliefs and encouraging the academic area, they get the feeling of self-study in children [**32**]. The mother’s occupation (89.4 percent housekeepers) had no statistically significance with any areas of self-efficacy.

## Conclusions

The results of this study confirmed the relation of assertiveness and self-efficacy in reducing or increasing aggression in students. In his study, Yousefi suggested that an increased awareness is associated with decreased aggression [**41**]. Debating and offering solutions, along with respecting the rights of others in the class discussions, could likely be a source of increasing assertiveness and self-efficacy and reducing aggression. One of the limitations of this research was the self-reporting tool. It suggested that in future studies, complementary methods such as observation and interviews should be used.

According to the findings of this study, it was suggested that in a future research, in addition to the variables mentioned above, one could examine the characteristics of the learning environment, schools-parents’ communication with students as well as students-peer communication.

**Acknowledgment**

The authors would like to thank all the principals and teachers of high schools, students and their parents who sincerely assisted in this study.
